# Rewired Cellular Metabolic Profiles in Response to Metformin under Different Oxygen and Nutrient Conditions

**DOI:** 10.3390/ijms23020989

**Published:** 2022-01-17

**Authors:** Shan Liu, Jumpei Washio, Satoko Sato, Yuki Abiko, Yuta Shinohara, Yuri Kobayashi, Haruki Otani, Shiori Sasaki, Xiaoyi Wang, Nobuhiro Takahashi

**Affiliations:** 1 Division of Oral Ecology and Biochemistry, Tohoku University Graduate School of Dentistry, Sendai 9808575, Japan; liu.shan.q3@dc.tohoku.ac.jp (S.L.); satoko.sato.b5@tohoku.ac.jp (S.S.); yuki.abiko.a8@tohoku.ac.jp (Y.A.); yuta.shinohara.b7@tohoku.ac.jp (Y.S.); yuri.kobayashi.c3@tohoku.ac.jp (Y.K.); haruki.ootani.t5@dc.tohoku.ac.jp (H.O.); shiori.sasaki.c1@tohoku.ac.jp (S.S.); nobuhiro.takahashi.a5@tohoku.ac.jp (N.T.); 2 Department of Head and Neck Oncology, Sichuan University West China School of Stomatology, Chengdu 610041, China; kqwxy@scu.edu.cn

**Keywords:** metformin, hypoxia, nutrient availability, rewired metabolic activity, oral cancer, dentistry

## Abstract

Metformin is a metabolic disruptor, and its efficacy and effects on metabolic profiles under different oxygen and nutrient conditions remain unclear. Therefore, the present study examined the effects of metformin on cell growth, the metabolic activities and consumption of glucose, glutamine, and pyruvate, and the intracellular ratio of nicotinamide adenine dinucleotide (NAD^+^) and reduced nicotinamide adenine dinucleotide (NADH) under normoxic (21% O_2_) and hypoxic (1% O_2_) conditions. The efficacy of metformin with nutrient removal from culture media was also investigated. The results obtained show that the efficacy of metformin was closely associated with cell types and environmental factors. Acute exposure to metformin had no effect on lactate production from glucose, glutamine, or pyruvate, whereas long-term exposure to metformin increased the consumption of glucose and pyruvate and the production of lactate in the culture media of HeLa and HaCaT cells as well as the metabolic activity of glucose. The NAD^+^/NADH ratio decreased during growth with metformin regardless of its efficacy. Furthermore, the inhibitory effects of metformin were enhanced in all cell lines following the removal of glucose or pyruvate from culture media. Collectively, the present results reveal that metformin efficacy may be regulated by oxygen conditions and nutrient availability, and indicate the potential of the metabolic switch induced by metformin as combinational therapy.

## 1. Introduction

Most tumors are rich in hypoxic and nutrient-insufficient regions generally because of the high rate of proliferation of their cells and poor vascularization. Oxygen concentrations range between 3 and 9.8% in normal tissues in a tissue type-dependent manner [1,2]. Oxygen concentrations are highly heterogeneous in human tumors because of different vascular distribution, tissue types, variable intratumor blood flow, or environmental stress [1,3,4,5]. Furthermore, metastatic cells scattered in the bloodstream may be exposed to high concentrations of oxygen.

Hypoxia is defined as an oxygen level that is lower than the value in the corresponding normal tissue [1]. Under hypoxic conditions, low oxygen concentrations may drive genetic instability, mutations, and metabolic reprogramming in tumor cells, which allows these cells to adapt to harsh environments and induces aggressive tumor phenotypes [2,6,7,8,9]. Hypoxia promotes tumor metastasis by enhancing epithelial-to-mesenchymal transition and correlates with poor survival in many cancers [10,11,12,13]. The development of resistance by tumor cells to conventional treatment, targeted therapy, and immunotherapy has been partly attributed to hypoxia [14,15,16]. Therefore, hypoxia is a crucial regulator of the progression and treatment of tumors. Insufficient nutrient availability is also a common phenotype in most cancers. Moreover, cancer cells survive in abnormal nutrient environments by regulating the gene expression of the tumor proteins P53, Myc, ATF4, and E74-like ETS transcription factor 2, interacting with mesenchymal cells or immune cells, and through metabolic adaptation [17]. Therefore, the nutrient environment may affect the efficacy of therapy. Previous studies reported that cytotoxic therapy exerted preferential anti-cancer effects in a nutrient-deficient environment, while other studies suggested that nutrient deficiency induced chemoresistance [18,19,20,21].

Metformin is a biguanide derivative and well-known first-line pharmacological treatment for type 2 diabetes [22,23]. The advantages of metformin for the treatment of type 2 diabetes include its effectiveness, robust safety, and low cost [23]. Apart from its beneficial effects on diabetes, accumulated evidence has demonstrated that metformin is a multifaceted drug that plays protective roles for inflammation, obesity, metabolic syndrome, cardiovascular diseases, endothelial function, cognitive decline, longevity, and even cancer [22,24,25,26]. Findings showing the possible contribution of metformin to reducing the risk of cancer in diabetes patients in 2005 was a prelude to the study of metformin in cancer prevention and treatment [23,27]. A group of clinical trials suggested that metformin showed well-tolerated safe profiles combined with or without traditional or targeted therapies in various cancers [28,29,30,31,32,33,34,35,36]. Clinical applications of metformin in cancer prevention and treatment recently indicated its promising therapeutic effects in colorectal cancer, breast cancer, non-small cell lung cancer, head and neck cancer, and other cancer types, whereas other studies found no beneficial effects in many cancers in non-diabetic patients [37,38,39]. The reason for these discrepant findings remains unclear, and attempts to stratify the best targets for metformin have not yet been conducted.

In laboratory studies, the inhibition of complex I, one of the essential components of the electron transport chain, by metformin was identified as a crucial mechanism for its anti-tumorigenic effects [40,41]. The ability of cells to restore metformin-induced disturbances is one of the most discussed factors affecting its efficacy [41,42]. The inhibition of electron transport chain functions by metformin may cause an imbalanced redox status, reduce energy production and biosynthesis, and disturb glucose and glutamine metabolism. Glucose and glutamine are two essential nutrients for rapid cell proliferation as the main carbon and nitrogen sources, respectively. Metformin has been shown to inhibit glycolysis or glutamine metabolism in breast cancer cells, hepatoma cells, oral cancer cells, and pancreatic cancer cells in order to suppress cell growth [43,44,45,46,47]. However, increased glycolysis and reductive glutamine metabolism have been proposed as metformin-resistant mechanisms in prostate cancer [48].

These contradictory findings indicate that the relationship between metformin efficacy and its metabolic responses remains unclear and, thus, further studies are warranted to promote its application to the treatment of cancer. Therefore, the present study investigated the anti-cancer effects of metformin under different oxygen and nutrient conditions as well as correlated metabolic profiles.

## 2. Results

### 2.1. Inhibitory Effects of Metformin on the Growth of Cancer and Normal Cells

**Figure 1:**
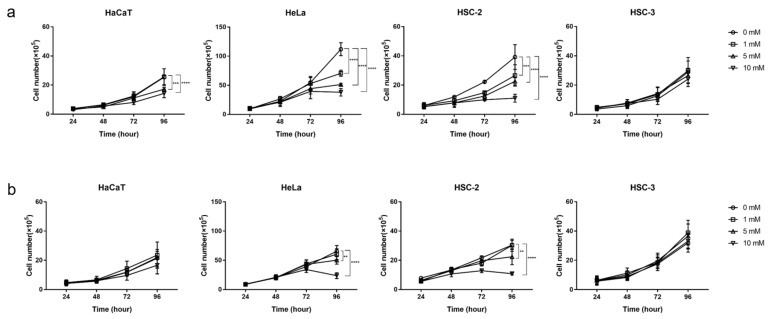
Time- and dose-dependent effects of metformin on cell growth. (**a**) Cells were cultured under normoxic conditions. (**b**) Cells were cultured under hypoxic conditions. A 2-way ANOVA and Tukey’s test. ** *p* < 0.01, *** *p* < 0.001, **** *p* < 0.0001. All experiments were independently performed in triplicate.

To investigate the anti-cancer effects of metformin under different oxygen conditions, cells were cultured with 0–10 mM metformin under normoxic (21% O_2_) and hypoxic (1% O_2_) conditions. Metformin exerted time- and dose-dependent effects on cell growth in HaCaT, HeLa, and HSC-2 (Figure 1). Under normoxic conditions, it significantly inhibited the growth of HaCaT, HeLa, and HSC-2 after 96 h (*p* < 0.01, Figure 1a); however, under hypoxic conditions, metformin did not significantly affect HaCaT growth and the inhibitory effects of 1 mM metformin on the growth of HeLa and HSC-2 were abrogated (Figure 1b). HSC-3 was resistant to metformin under normoxic and hypoxic conditions (Figure 1a,b).

### 2.2. Acute Effects of Metformin on Cellular Metabolic Activity

**Figure 2:**
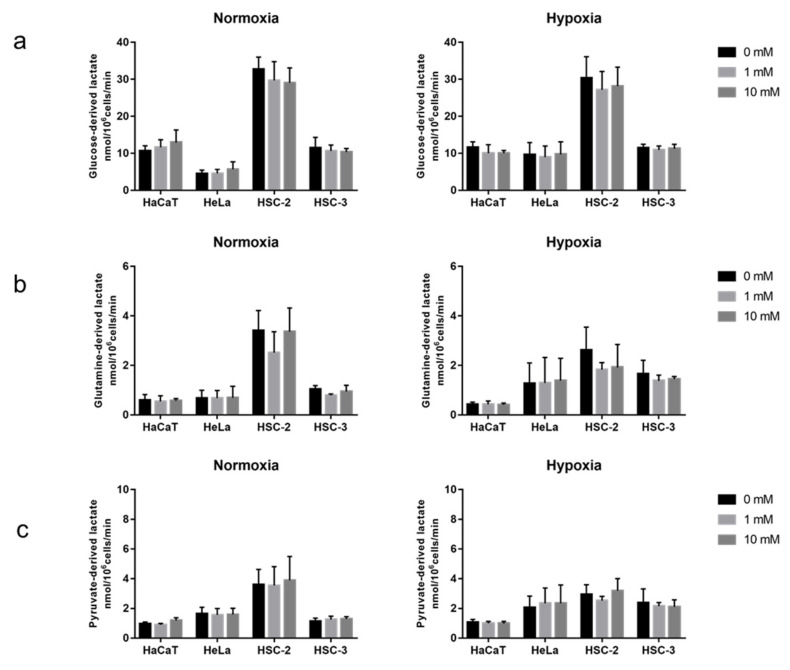
Instant effects of metformin on cellular metabolic activity. (**a**) Glucose-derived lactate in normoxia (**left**) and hypoxia (**right**). (**b**) Glutamine-derived lactate in normoxia (**left**) and hypoxia (**right**). (**c**) Pyruvate-derived lactate in normoxia (**left**) and hypoxia (**right**). A one-way ANOVA and Tukey’s test. All experiments were independently performed in triplicate.

To detect the instant effects of metformin on different fuel metabolic activities, cells were treated with a single nutrient and 0–10 mM metformin for 1 h. The results obtained show that metformin did not significantly affect glucose-, glutamine-, or pyruvate-derived lactate production under normoxic or and hypoxic conditions (Figure 2).

### 2.3. Effects of Long-Term Exposure to Metformin on Cellular Nutrient Utilization and Organic Acid Production in Culture Media

**Figure 3:**
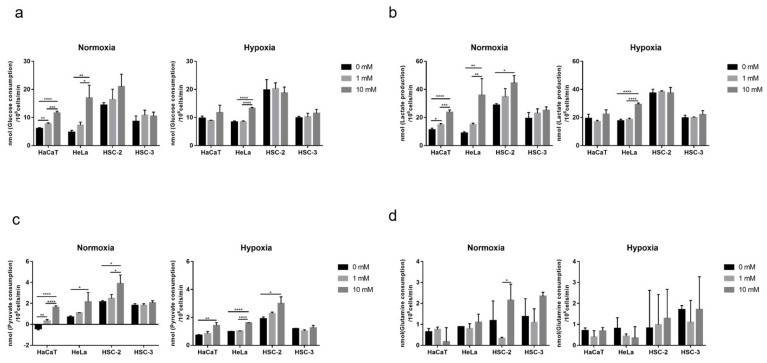
Nutrient consumption during growth with metformin under normoxia and hypoxia. (**a**) Glucose consumption in normoxia (**left**) and hypoxia (**right**). (**b**) Lactate production in normoxia (**left**) and hypoxia (**right**). (**c**) Pyruvate consumption in normoxia (**left**) and hypoxia (**right**). (**d**) Glutamine consumption in normoxia (**left**) and hypoxia (**right**). A one-way ANOVA and Tukey’s test. * *p* < 0.05, ** *p* < 0.01, *** *p* < 0.001, **** *p* < 0.0001. All experiments were independently performed in triplicate.

All cells exhibited a preference for glucose over glutamine and pyruvate in complete medium (Figure 3). Despite the growth inhibitory effects of metformin (Figure 1), glucose consumption in HaCaT, HeLa, and HSC-2 was slightly increased by long-term exposure (72–96 h) to metformin in a dose-dependent manner under normoxic conditions. The long-term exposure to metformin significantly enhanced glucose consumption in HaCaT and HeLa under normoxic conditions, and this was reduced by hypoxia (Figure 3a). Lactate production showed similar responses to glucose consumption in the presence of metformin (Figure 3b). A high concentration of metformin enhanced pyruvate consumption in HaCaT, HeLa, and HSC-2 under normoxic and hypoxic conditions (Figure 3c). Metformin did not significantly affect glucose consumption, lactate production, or pyruvate consumption in HSC-3 regardless of oxygen conditions (Figure 3a–c). Furthermore, significant differences were observed in the effects of low and high concentrations of metformin on glutamine consumption in HSC-2 under normoxic conditions (Figure 3d). Metformin did not significantly affect glutamine consumption in HaCaT, HeLa, or HSC-3.

### 2.4. Effects of Long-Term Exposure to Metformin on Cellular Metabolic Activity

**Figure 4:**
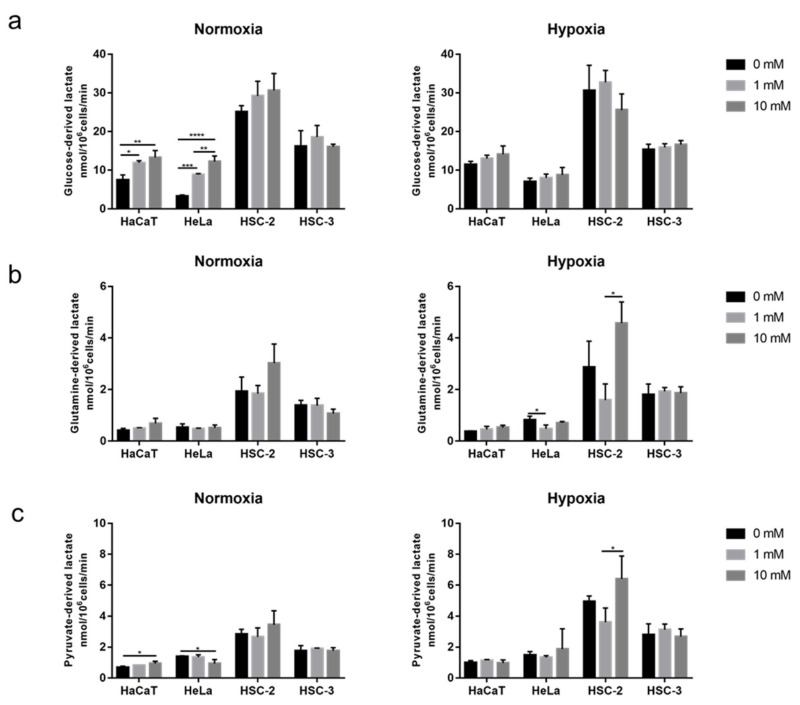
Modification of metabolic profiles during growth with metformin under normoxia and hypoxia. (**a**) Glucose-derived lactate in normoxia (**left**) and hypoxia (**right**). (**b**) Glutamine-derived lactate in normoxia (**left**) and hypoxia (**right**). (**c**) Pyruvate-derived lactate in normoxia (**left**) and hypoxia (**right**). A one-way ANOVA and Tukey’s test. * *p* < 0.05, ** *p* < 0.01, *** *p* < 0.001, **** *p* < 0.0001. All experiments were independently performed in triplicate.

We used a single nutrient as the substrate to detect different fuel metabolic activities. Glucose-derived lactate production significantly increased in a dose-dependent manner in HaCaT and HeLa grown with metformin under normoxic conditions (Figure 4a). Under hypoxic conditions, glucose-derived lactate production showed no response to metformin in HaCaT and HeLa (Figure 4a). Furthermore, no significant differences were observed in glucose-derived lactate production between HSC-2 and HSC-3 under normoxic and hypoxic conditions (Figure 4a).

Glutamine was utilized and converted to lactate by glutaminolysis. No significant differences were noted in glutamine-derived lactate production induced by the long-term exposure to metformin in these four cell lines under normoxic conditions (Figure 4b). However, under hypoxic conditions, glutamine-derived lactate was significantly inhibited in HeLa by the low concentration of metformin, while a significant difference was observed between low and high concentrations of metformin in HSC-2. The high concentration of metformin resulted in greater glutamine-derived lactate production than the low concentration (Figure 4b).

Pyruvate was used to examine the effects of metformin on the pathway from pyruvate to lactate. The high concentration of metformin significantly enhanced pyruvate-derived lactate production in HaCaT, but inhibited it in HeLa under normoxic conditions (Figure 4c). Pyruvate-derived lactate production in HSC-2 and HSC-3 was not affected by metformin under normoxic conditions (Figure 4c). Under hypoxic conditions, a significant difference was observed between the low and high concentrations of metformin in HSC-2. The high concentration of metformin resulted in greater pyruvate-derived lactate production than the low concentration (Figure 4c).

Overall, glucose was the main substrate for cellular metabolic activity in all types of cells investigated in the present study. Other substrates, glutamine and pyruvate, were not efficiently utilized under the conditions provided with culture media.

### 2.5. Decreased Cellular NAD^+^/NADH Ratio during Cell Growth with Metformin

**Figure 5:**
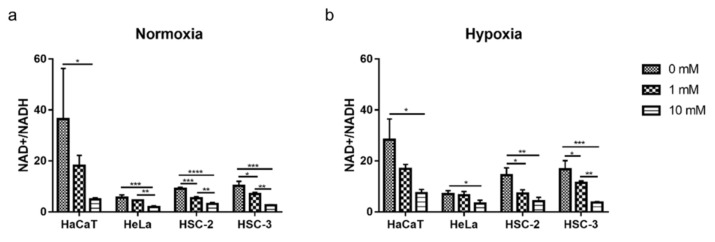
Decreased NAD^+^/NADH ratio during growth with metformin under normoxia (**a**) and hypoxia (**b**). A one-way ANOVA and Tukey’s test. * *p* < 0.05, ** *p* < 0.01, *** *p* < 0.001, **** *p* < 0.0001. All experiments were independently performed in triplicate.

Metformin decreased the NAD^+^/NADH ratio during cell growth, and this effect was significant at 10 mM in the four cell lines under both normoxic and hypoxic conditions (Figure 5).

### 2.6. Effects of Nutritional Conditions on Metformin Efficacy

**Figure 6:**
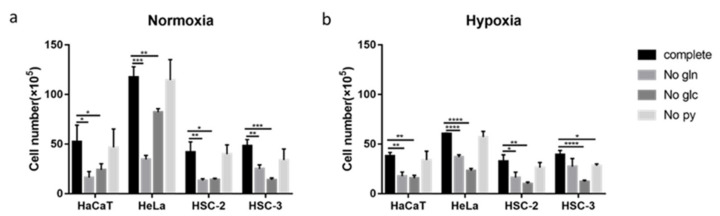
Effects of nutrient conditions on cell growth. (**a**) Effects of glucose, glutamine, and pyruvate on cell growth under normoxia. (**b**) Effects of glucose, glutamine, and pyruvate on cell growth under hypoxia. The Student’s *t*-test. * *p* < 0.05, ** *p* < 0.01, *** *p* < 0.001, **** *p* < 0.0001. All experiments were independently performed in triplicate. “Complete” means cells cultured in DMEM with all components, “No gln” means cells cultured in glutamine-free DMEM, “No glc” means cells cultured in glucose-free DMEM, and “No py” means cells cultured in pyruvate-free DMEM.

The growth of the four cell lines was significantly inhibited by the removal of glucose or glutamine from complete medium regardless of the oxygen condition, except for HSC-3 in glutamine-free medium under hypoxic conditions (Figure 6a,b). The growth of HSC-3 was significantly suppressed in pyruvate-free medium under hypoxic conditions (Figure 6b). Therefore, the effects of metformin on cell growth under different nutrient conditions were examined.

**Figure 7:**
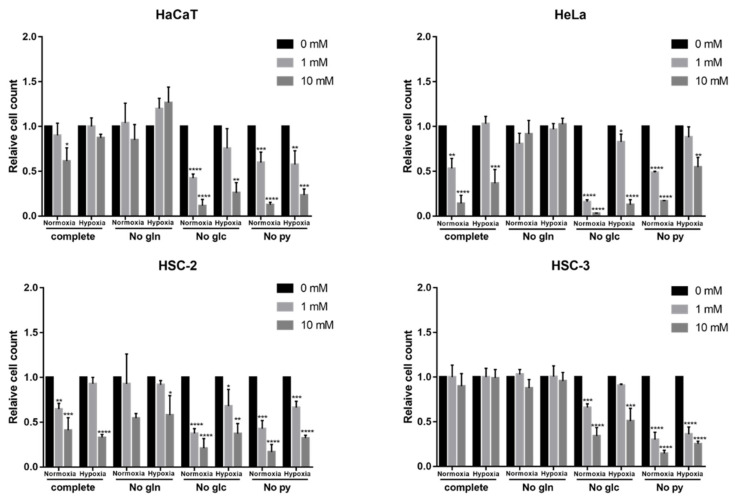
Effects of nutrient removal on metformin efficacy. Sensitivities of cell lines to metformin in complete medium (complete), glutamine-free medium (No gln), glucose-free medium (No glc), and pyruvate-free medium (No py) under normoxia and hypoxia. The relative cell count indicates the metformin group/control group. A one-way ANOVA and Tukey’s test. * *p* < 0.05, ** *p* < 0.01, *** *p* < 0.001, **** *p* < 0.0001. All experiments were independently performed in triplicate.

In glucose-free medium, metformin significantly inhibited the proliferation of all cells, including HSC-3, especially under normoxic conditions (Figure 7). In pyruvate-free medium, metformin also significantly inhibited the growth of all cells and was more sensitive under normoxic conditions (Figure 7). These growth inhibitory effects of metformin were more potent than those in complete medium. However, metformin-induced reductions in the growth of these four cell lines were attenuated in glutamine-free medium, and the inhibitory effects of metformin were almost abolished in HaCaT and HSC-2 (Figure 7).

## 3. Discussion

**Figure 8:**
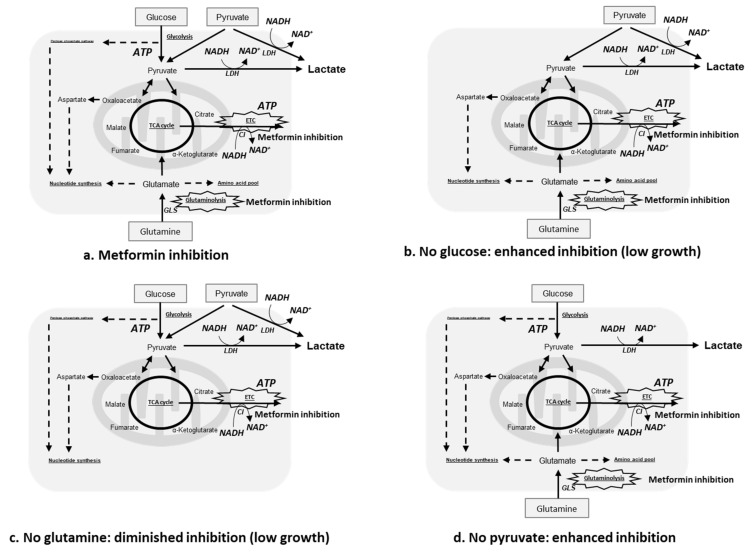
Proposed metabolic rewiring of the metabolism of glucose, glutamine, and pyruvate in response to metformin inhibition. Metformin inhibition in (**a**) complete, (**b**) glucose-free (No glucose), (**c**) glutamine-free (No glutamine), or (**d**) pyruvate-free (No pyruvate) medium. ETC, electron transport chain; LDH, lactate dehydrogenase; CI, complex I; GLS, glutaminase.

Hypoxia attenuated the growth inhibitory effects of metformin in HaCaT, HeLa, and HSC-2 (Figure 1), suggesting that the concentration of oxygen is one of the factors affecting its efficacy. In a previous study on breast cancer cells, metformin efficacy was also decreased by hypoxia [49]. Recent findings revealed that the stimulatory mechanism for the anti-cancer effects of metformin involves its inhibition of the electron transport chain (Figure 8a), mainly complex I, in many cancers, such as colon cancer, lung cancer, breast cancer, cervical adenocarcinoma, and bone osteosarcoma [40,41]. The electron transport chain is suppressed under hypoxic conditions, which reduces dependence on oxidative phosphorylation (OXPHOS) for cell proliferation, potentially through a decrease in metformin efficacy under these conditions. However, the present study also showed that HaCaT, a normal cell line with a priority to use OXPHOS, was less sensitive to metformin than HeLa and HSC-2 under normoxic conditions (Figure 1). These results suggest that a dependence on OXPHOS was not the sole factor affecting metformin efficacy in these cells. These cell lines showed heterogeneous sensitivities to metformin, which might be due to a cell’s intrinsic phenotypes, derived from the expression of metformin transporters and mitochondrial mutations [41,42], affecting metformin efficacy. Nevertheless, we also found that metformin modified the cellular utilization of carbon sources (Figure 3 and Figure 4), while the availability of carbon sources altered the sensitivities of these cells to metformin (Figure 6 and Figure 7) under different oxygen conditions. These results suggest that the above-described intrinsic factors were not determinants for metformin efficacy in the cells used in the present study; metabolic rewiring has been more closely associated with heterogeneous cell responses to metformin [50]. Collectively, the present results identified oxygen and environmental carbon sources as factors affecting metformin efficacy and argue that metabolic rewiring is crucial for cell sensitivity to metformin under different oxygen and nutrient conditions, as discussed in detail below.

The long-term exposure to metformin induced a cellular metabolic switch (Figure 2 and Figure 4). Despite the growth inhibitory effects of metformin (Figure 1), HaCaT, HeLa, and HSC-2 increased their glucose metabolic activities during the long-term exposure to metformin, particularly under normoxic conditions (Figure 4a). As discussed above, metformin inhibits complex I in the electron transport chain (Figure 8a), resulting in an ATP shortage through a functional disorder in OXPHOS. To rescue this condition, these cells appeared to promote glycolysis for ATP production, as suggested by increases in lactate production in the presence of metformin (Figure 3b). Pyruvate was also utilized more in the presence of metformin (Figure 3c), indicating that pyruvate supports the conversion of NADH to NAD^+^ (Figure 8a) because maintaining a high NAD^+^/NADH ratio is essential for smooth metabolism, particularly under the inhibition of complex I in which NADH inevitably accumulates. Additionally, the increased consumption of pyruvate may be employed for a reverse exchange flux in the TCA cycle to rescue mitochondrial function for biosynthesis [42]. Metformin-resistant cells and metformin-sensitive cells displayed a stable flux of glycolysis and pyruvate consumption under hypoxic conditions regardless of the NAD^+^/NADH state (Figure 3a,c, Figure 4a,c and Figure 5). Therefore, metformin appeared to induce a disturbance in NAD^+^ and NADH regardless of its growth inhibitory effects, whereas metformin-resistant cells sustained cell proliferation, but not by enhancing glycolysis and pyruvate consumption. Metformin-resistant cells may be able to adapt nutrient utilization in the mitochondria by up-regulating OXPHOS gene expression and enhancing reductive glutamine metabolism, pyruvate carboxylase activity, or glutamate oxaloacetate transferase activity [42,48,51,52]. These findings indicate that metabolic plasticity induced by metformin was dependent on the cell lines examined and oxygen conditions, suggesting that the metabolic regulation of glycolysis, glutaminolysis, and pyruvate utilization by a metformin disturbance involved a complex network of genetic and environmental factors. Previous studies reported that metformin suppressed the activities of the key enzymes, hexokinase, pyruvate kinase M2, and phosphofructokinase 1 in glycolysis as well as glutaminase (GLS) in glutaminolysis, which contributed to its growth inhibitory effects [44,47,53,54]. In the present study, by evaluating the metabolic activities of glycolysis and glutaminolysis in cells grown in the presence of metformin (Figure 3 and Figure 4) and the inhibitory effects of metformin on cell growth in media excluding each metabolic substrate (Figure 6 and Figure 7), the extent to which individual metabolic systems as a whole contribute to the inhibition of cell growth by metformin was clarified.

Although the sensitivities of the four cell lines to metformin were diverse under normoxic and hypoxic conditions, as discussed above, metformin decreased the NAD^+^/NADH ratio regardless of the cell line or oxygen condition (Figure 5). These results support metformin inhibiting complex I in the mitochondrial electron transport chain, but also suggest that the NAD^+^/NADH ratio is not an independent marker for metformin efficacy even though Gui et al. reported that changes in the NAD^+^/NADH ratio correlated with the growth inhibitory effects of metformin [41]. Difficulties are associated with identifying an independent metabolic predictor for metformin efficacy based on recent findings. Accumulated evidence suggests that the ability of cancer cells to sustain aspartate levels is useful for predicting metformin efficacy; however, Liu et al. found that aspartate levels were not an independent predictive marker for metformin efficacy in ovarian cancer [42,52]. Furthermore, reductive glutamine metabolism may be enhanced in prostate cancer cells and autophagy activity may be up-regulated in cholangiocarcinoma cells to resist the cytotoxicity of metformin [48,55]. Collectively, these findings indicate that tumor heterogeneity is important for metformin responses and combinatorial research is needed to obtain a more detailed understanding of these responses in the future.

A shift towards glycolysis- and/or glutaminolysis-based energy production has been regarded as one of the mechanisms underlying metformin resistance in many cancers, with pyruvate derived from glycolysis and/or glutaminolysis being converted to lactate by lactate dehydrogenase with the consumption of NADH (Figure 8a). However, the present results show that increases in lactate production did not always occur, particularly under hypoxic conditions and in metformin-resistant cells (Figure 4), which may be attributed to the difficulties associated with differentiating between glycolysis- and glutaminolysis-based lactate production. In other words, even if glycolysis-based lactate production is enhanced, total lactate production will not increase if glutaminolysis-based lactate production is suppressed, as discussed below. Furthermore, the profile of metformin on lactate production in hypoxia supported the application of metformin in patients with hypoxemia, which was once considered a contraindication to metformin [56].

Changes in sensitivity to metformin in non-complete media (Figure 6 and Figure 7) may be due to metabolic rewiring or the inability to sustain redox and nutrient balances. The increased sensitivity of cell proliferation to metformin observed in glucose-free medium (Figure 7, No Glc) may be associated with the effects of metformin on glutamine metabolism (Figure 8b); the dependence on glutamine metabolism for anabolic metabolism may be enhanced when cells are cultured under glucose-free conditions [57] and the proliferation of glutamine-dependent cells becomes sensitive to metformin [58]. Metformin has been reported to impair glutamine metabolism by inhibiting GLS, an initial metabolic enzyme for the degradation of glutamine [47]. In glutamine-free medium, the metformin-induced inhibition of cell growth was diminished (Figure 7, No Glu), possibly because glutamine metabolism, one of the metabolic pathways sufficiently inhibited by metformin as described above, became unfunctional (Figure 8c). Exogenous pyruvate may be utilized to maintain the NAD^+^/NADH ratio and aspartate levels in order to counteract the effects of metformin (Figure 8a) [41,59]. The increased sensitivity to metformin in pyruvate-free medium (Figure 7, No Pyr) may be attributed to the lack of the functions provided by pyruvate (Figure 8d). Since metformin inhibits the electron transfer system, resulting in the accumulation of NADH, the conversion of NADH to NAD^+^ using an oxidizing substrate, such as pyruvate, appears to be essential for maintaining smooth cell metabolism, as discussed above.

The present study identified possible factors for metformin efficacy and indicated that metformin induced heterogeneous metabolic profiles in different cell lines, which was affected by oxygen conditions. These results support the emerging view that cancer metabolism is flexible and context-specific or changes according to environmental conditions [60]. Metabolic heterogeneity is a common, but sophisticated, profile in tumors and is induced by genetic alterations, epigenetic modifications, and microenvironment factors [61]. The present results support these findings and, thus, metabolic macroscopic parameters do not appear to be independent indexes for metformin predictive targets. At the same time, by limiting metabolic substrates, such as removing glucose or pyruvate from the culture medium, the sensitivity of all cells to metformin increased, including cells with metformin-resistant properties, such as HSC-3 (Figure 7, No Glu and No Pyr). This increase in metformin efficacy by metabolic profile rewiring will facilitate the development of more effective clinical applications for metformin in cancer treatment.

## 4. Materials and Methods

### 4.1. Chemical Regents

Metformin (136-18662), glucose (049-31165), L-glutamine (074-00522), sodium pyruvate (199-03062), and sodium pyruvate solution (190-14881) were purchased from Fujifilm-Wako (Fujifilm-Wako Pure Medical Corporation, Miyazaki, Japan).

### 4.2. Cell Lines and Culture Conditions

The human oral squamous cell carcinoma cell lines, HSC-2 (JCRB0622: oral squamous carcinoma cells derived from weak gingivae) and HSC-3 (JCRB0623: oral squamous carcinoma cells derived from highly advanced tongue cancer) (JCRB Cell Bank, Tokyo, Japan), human cervical adenocarcinoma cells (HeLa) (TKG0331, Cell Resource Center for Biomedical Research in Tohoku University, Miyagi, Japan), and the normal human keratinized epithelial cell line, HaCaT (Cosmo Bio Co., Ltd., Tokyo, Japan), were used in the present study. All cell lines were authenticated using a short-tandem repeat analysis. All cells were cultured in low glucose Dulbecco’s modified Eagle’s medium (DMEM) (041-29775, Fujifilm-Wako Pure Medical Corporation, Miyazaki, Japan) supplemented with 10% heat-inactivated fetal bovine serum (Life Technologies, New York, NY, USA), 100 mg/mL streptomycin, and 100 U/mL penicillin (Fujifilm-Wako Pure Medical Corporation, Miyazaki, Japan) at 37 °C in a humidified atmosphere with 5% CO_2_.

### 4.3. Cell Proliferation Assays

Cells were passaged at the logarithmic stage, and 2.5 × 10^5^ cells were seeded on 100-mm culture plates overnight. Medium was changed to treatment medium with 0–10 mM metformin and cells were cultured in 21% O_2_ (normoxia) and 1% O_2_ (hypoxia) incubators. Cell numbers were automatically counted by Countless TM II FL (Thermo Fisher Scientific, Waltham, MA, USA) at the indicated time points (24, 48, 72, and 96 h).

To assess the effects of nutrient removal on metformin efficacy, cells were cultured in DMEM with all components (041-29775, Fujifilm-Wako Pure Medical Corporation, Miyazaki, Japan), DMEM without glutamine (044-33555, Fujifilm-Wako Pure Medical Corporation, Miyazaki, Japan), DMEM without glucose, and DMEM without pyruvate (042-32255, Fujifilm-Wako Pure Medical Corporation, Miyazaki, Japan) under different oxygen conditions after cell adherence. Metformin (0, 1, and 10 mM) was used to treat cells. Cell numbers were automatically counted by Countless TM II FL (Thermo Fisher Scientific, Waltham, MA, USA) at 96 h.

### 4.4. Detection of Metabolic Activities of Glucose, Glutamine, and Pyruvate in Cells with or without an Acute Exposure to Metformin

Cells (2.5 × 10^5^) were seeded on 100-mm culture plates overnight and allowed to grow to the logarithmic stage. Cells on culture dishes were gently washed with PBS twice, and 5 mM glucose, glutamine, or pyruvate solution was then added to culture plates with metformin (0–10 mM). After incubation for 1 h, the reaction solution was recovered and immediately stored at −80 °C. Cell numbers were counted with an automatic cell counter. All samples were analyzed by high-performance liquid chromatography (HPLC) to detect the secretion of organic acids as the end products of metabolism.

### 4.5. Measurement of Concentrations of Pyruvate, Glucose, and Glutamine in Culture Media

Cells (2.5 × 10^5^) were seeded on 100-mm culture plates overnight and allowed to grow to the logarithmic stage with 0, 1, and 10 mM metformin in complete medium. Treatment medium was changed every 24 h to maintain a stable nutrient supply. Culture media were collected after 72 h for HSC-2, HSC-3, and HeLa and after 96 h for HaCaT, and nutrient consumption was evaluated. Glucose concentrations were assessed using the Glucose C2 test (134-19121, Fujifilm-Wako Pure Medical Corporation, Miyazaki, Japan) and glutamine concentrations by the Glutamine Assay Kit-WST (G268, Dojindo, Kumamoto, Japan) according to the manufacturers’ instructions. Pyruvate concentrations were evaluated by HPLC.

### 4.6. Detection of Metabolic Activities of Glucose, Glutamine, and Pyruvate in Cells Grown with or without Long-Term Exposure to Metformin

Cells (2.5 × 10^5^) were seeded on 100-mm culture plates overnight and allowed to grow to the logarithmic stage with metformin (0–10 mM). Cells on culture dishes were gently washed with PBS twice, and 5 mM glucose, glutamine, or pyruvate solution was then added to culture plates with metformin. After incubation for 1 h, the reaction solution was recovered and immediately stored at −80 °C. Cell numbers were counted with an automatic cell counter. All samples were analyzed by HPLC to detect the production of organic acids as the end products of metabolism.

### 4.7. Analysis of Organic Acids with HPLC

The samples collected from the above-described experiments were analyzed for organic acids using HPLC (Prominence LC-20AD, Shimadzu Corporation, Kyoto, Japan).

### 4.8. NAD^+^/NADH Ratio Assay

Cells (2.5 × 10^5^) were seeded on 100-mm culture plates overnight and allowed to grow to the logarithmic stage with or without metformin (0–10 mM). Cells were then washed with PBS and used in the NAD/NADH assay Kti-WST (N509, Dojindo, Kumamoto, Japan) to extract and detect NAD^+^ and NADH concentrations according to the manufacturer’s instructions.

### 4.9. Statistical Analysis

The Student’s *t*-test was used to analyze the significance of differences between two groups. A one-way ANOVA was performed to examine the difference by one factor in multiple groups. A two-way ANOVA was conducted to assess the difference by two factors in multiple groups. Results were expressed as the mean ± SD (standard deviation). *p* values of <0.05 were considered to be significant (StatFlex, version 6).

## 5. Conclusions

The metabolic profiles of cells were closely related to their sensitivities to metformin. The present study demonstrated that metformin sensitivity was regulated by the cellular metabolic profile, which was rewired by oxygen conditions and the nutrient supply. These results will contribute to the development of effective clinical applications for metformin.

## Data Availability

The data that support the present results are available from the corresponding author upon reasonable request.
